# Treatment of Teeth Rendered Sensitive by Mechanical Abrasion

**Published:** 1868-05

**Authors:** 


*Answers to Querists.*

*Query 1st.*—"What treatment promises most success for the relief of that tenderness in teeth that are much worn?"

*Answer.*—For temporary relief from the effects of *mechanical abrasion*, where this abrasion has progressed so far as to render the tooth susceptible to impressions of heat and cold, the contact of acids, &c., either of the following applications may be made to the surface of the dentine immediately over the pulp cavity: chromic acid, chloride of zinc, terchloride of gold, or carbolic acid.

For permanent relief, especially where the irritation has been so great as to cause inflammation of the pulp, it becomes necessary for the preservation of the tooth to destroy this organ. To accomplish this the following method may be pursued: no cavity existing in these cases for the retention of such agents as arsenious acid combined with sulph. morphia and creosote, we proceed to make an opening into the pulp cavity through the thin layer of dentine immediately over it, this layer forming what is, or has become, in the case of a front tooth, the grinding surface. As the application of the drill to to such a surface would cause intense and protracted pain, the spray apparatus may be used effectually upon it before applying the drill. When the anæsthetic effect is complete, the drill can be used with impunity, and, as it approaches the pulp, should reaction take place the spray may again be applied and in this manner the pulp be exposed. When an opening has been thus made to the pulp, a barbed nerve instrument may be used for removing it entire, taking care to again apply the spray immediately before the barbed instrument is thrust into the canal. The pain which would follow the application of the spray directly to the exposed pulp, may, in a measure at least, be lessened by first introducing a small pellet of cotton into the opening made by the drill. We prefer, in such cases, removing the pulp entire, by means of a barbed nerve instrument, to the application of arsenious acid, believing that there is a greater chance for the preservation of the tooth by this method. When the entire pulp has been thus removed, the cavity should be syringed thoroughly with tepid water, a small pellet of cotton saturated with creosote or carbolic acid introduced into the pulp cavity and carried near to the foramen at the apex of the root, when the canal is ready for a filling of gold, or such material as may be preferred for this purpose.

Another method for obtunding the sensibility of the surface upon which the drill is to be used in order to expose the pulp, is to apply nitric acid by means of a small pointed stick of wood, taking care that the acid does not come in contact with any other part than that in which the opening is to be made. As the drill makes its way towards the pulp cavity, should sensitiveness be experienced, the application of the acid may be repeated, and in this manner the pulp be exposed with comparatively little pain to the patient.

